# Do depression and its associated factors differ in women daytime and shift workers?: an analysis of the Korea National Health and Nutrition Examination Survey 2018

**DOI:** 10.4069/kjwhn.2021.05.17

**Published:** 2021-06-14

**Authors:** Hyun Ju Chae, Mijong Kim

**Affiliations:** 1 Department of Nursing, Joongbu University, Geumsan, Korea; 2 Department of Nursing, Hannam University, Daejeon, Korea

**Keywords:** Depression, Health behavior, Health services, Shift work schedule, Working women

## Abstract

**Purpose:**

This study examined health behaviors, use of health services, and depression among women who perform daytime and shift work in Korea, as well as factors related to depression.

**Methods:**

We conducted a secondary analysis using data from the 2018 data of the 7th Korea National Health and Nutrition Examination Survey. Data on women, 1,493 regular daytime workers and 322 shift workers, were analyzed.

**Results:**

Women shift workers were younger (χ^2^=43.97, *p*<.001), had a lower education level (χ^2^=45.56, *p*<.001), and lower personal income (χ^2^=16.85, *p*=.030) than their daytime counterparts. A higher proportion of shift workers were unmarried (χ^2^=37.47, *p*<.001) and they typically worked fewer than 40 hours per week (χ^2^=69.94, *p*<.001). The depression score of shift workers was higher than that of daytime workers (t=2.85, *p*=.005). A higher proportion of shift workers also drank alcohol (χ^2^=6.49, *p*=.032) and smoked (χ^2^=30.79, *p*<.001). Over 8% of shift workers typically slept fewer than 5 hours per night (χ^2^=14.17, *p*=.024). It was confirmed that depression in women shift workers was affected by age, personal income, marital status, health status, and smoking status, in addition to cancer screening participation, unmet medical care needs, and unmet dental care needs.

**Conclusion:**

More attention should be given to the health needs of women working shifts. Health promotion programs specific for women shift workers are needed to improve their physical and mental health, encourage use of medical care services, and improve public health policies and systems.

## Introduction

As the desire for higher education and self-actualization has increased among women in Korea, their participation in the economy has also increased, with the rate of employment for women rising from 54.9% in 2011 to 60.0% in 2019 [1,2]. However, this remarkable increase in women’s employment rate does not mean that women’s self-actualization and quality of life have necessarily improved. This is because a high proportion of women undertake non-regular shift work and part-time work compared to men, and inequality has been identified as a new problem related to the different employment rates and employment patterns of men and women [3]. Married women in the workforce in particular, must participate in economic activities on a par with men, while also performing additional household roles such as housekeeping and childcare; these coexisting demands can be considered hazardous to their health [1,4].

Shift work refers to a type of work in which people are divided into two or more groups, and the daily work is divided into two or more shifts [5,6]. With the growing emphasis on convenience in everyday life, shift work has been increasing in popularity in recent years [5], and there has been a growing interest in the impact of shift work on workers’ physical, mental, and social health.

Compared to regular daytime workers, shift workers are more likely to experience disruptions in their daily routines and lives due to irregular working hours, and thus have more difficulty maintaining positive everyday health behaviors [7,8]. It is well-known that shift work interferes with workers’ normal sleep-wake cycles, especially night shift workers, for whom the risk of safety accidents is high due to decreased concentration. Thus, a high proportion of shift workers in general tend to engage in unhealthy behaviors such as not getting enough sleep, smoking, and drinking alcohol [8,9]. As a result, shift work can lead to difficulties maintaining good health in daily life and increase workers’ vulnerability to diseases. Shift workers frequently experience digestive, musculoskeletal, metabolic, and cerebrovascular diseases [8-10], and women shift workers have been reported to have an elevated risk of breast cancer [9,10] and premature birth [10] or miscarriage [10].

Studies on the mental health of shift workers have found that shift workers tend to experience a variety of mental health problems such as high stress [11], depression, and anxiety [7]. Serious depression among women shift workers was also found in preceding studies [11,12]. According to the Korean National Health and Nutrition Examination Survey (KNHANES), women shift workers were 2.94 times more likely to develop depression than their male counterparts [12], and the degree of depression experienced by women shift workers was 1.73 times higher than men according to a meta-analysis of research on shift workers [11]. However, previous studies have not provided sufficient evidence regarding why women shift workers experience more serious depression than men shift workers. Some studies have suggested that women generally have a higher risk of depression than men [11], while others have suggested that hormonal reactions can cause women to be more stressed about rotated or shift work, making them more vulnerable to depression than men [12]. One explanation for the high levels of depression among women shift workers may be that childcare or housework duties increase their fatigue and stress levels in addition to the negative physical and mental effects of shift work [1,3]. In order to identify the causes and factors that influence depression among women shift workers, studies that are designed to exclude the effects of exogenous variables should be conducted.

Unmet medical care needs, which occur when people are unable to use medical services despite experiencing illness or health problems that require medical attention, is an important direct indicator of medical inequality [13,14]. These variables are meaningful indicators of the health of women who perform shift work. According to an analysis of adult women in Korea, the extent to which women received insufficient medical care was at least 1.2 times and up to 1.5 times higher for employed women than for unemployed women, and the reasons for insufficient medical care among women were costs, long wait times at doctor’s offices, difficulties obtaining health services during working hours, and childcare responsibilities [15]. For employed women, analysis of unmet medical conditions of daytime workers and shift workers with modified working schedules is sorely lacking and more in-depth research is needed.

Prior studies have found that shift work and depression are related. Although women shift workers reported more serious depression than men shift workers, most studies on depression that included women shift workers classified all participants simply as shift workers, not accounting for gender differences in their analyses [11,12], or examined health behaviors, depression, and quality of life among all women workers regardless of work type [1,15]. In many studies, women shift workers were not distinguished from other women workers according to their working patterns. In addition, studies of depression among shift workers have typically compared workers by sex, and many of the interpretations resulting from these studies have been fragmented [11,12].

Among women workers, those who perform shift work tend to face many health vulnerabilities. Studies that examine women workers should further classify them as daytime or shift-based workers, according to the type of work performed, in order to more accurately identify differences in health status and depression. Furthermore, in order to better understand women shift workers’ health, identifying their unmet medical needs, which tend to be indicators of general characteristics, health behaviors, and medical inequality, is also of value. Depression among women shift workers has already been shown to be at a serious level [11,12]. Thus, it is important and urgent to identify the factors affecting depression among women shift workers as a next step.

This study was therefore conducted to examine shift working women’s health behaviors, health care use, and depression according to their type of work, and analyze factors related to depression. This would improve the existing understanding of women shift workers’ mental health, and enable suggestions for future health promotion programs targeted to women shift workers, and provide basic data to support policies that address their needs.

### Purpose of the study

This study was conducted to examine health behaviors, the use of medical services, and depression among women daytime workers and shift workers, and to identify factors related to depression using the 2018 KNHANES data. The specific objectives of this study were as follows.

• To identify and compare the general characteristics, health-related behaviors, use of medical services, and the prevalence of depression among women daytime and shift workers

• To analyze differences in the prevalence and degree of depression according to the general characteristics, health-related behaviors, and use of medical services of female daytime and shift workers

• To analyze factors related to depression among female daytime and shift workers

## Methods

Ethics statement: This study was exempted by the Institutional Review Board (IRB) of Hannam University (IRB-2020-E-03-01). This study was a secondary analysis of data and data were received anonymously.

### Design

This secondary analysis study used 2018 data from the 7th KNHANES (2016-2018), with a correlational research design, to compare differences in health-related behaviors, medical service usage, and depression among working women and to identify factors related to depression among working women. This study was described in accordance with the Strengthening the Reporting of Observational Studies in Epidemiology (STROBE) guidelines (https://www.strobe-statement.org/index.php?id=strobe-home).

### Participants

**Figure 1.**
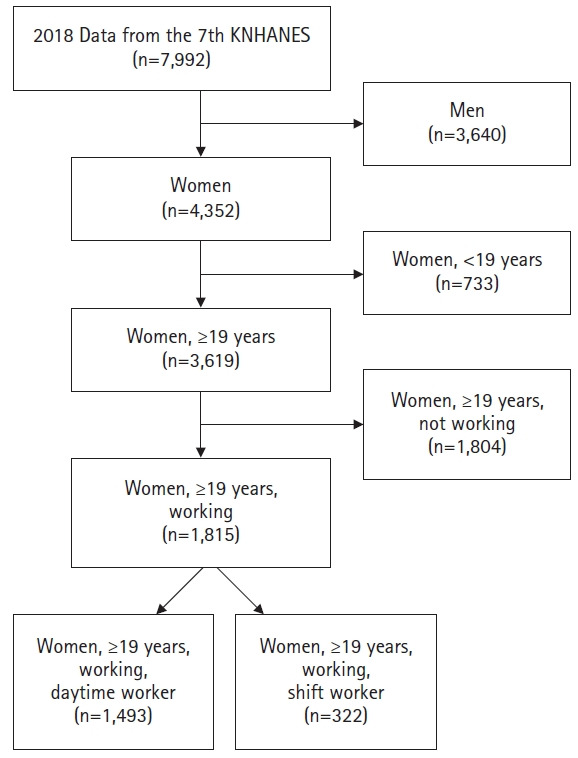
Flowchart of the study population. KNHANES: Korea National Health and Nutritional Examination Survey.

Participants were working women aged 19 years or older who participated in the 7th KNHANES, during the period of January to December 2018, conducted by the Korea Centers for Disease Control and Prevention. Women were considered employed if they answered “yes” to the following question: “Have you worked for more than 1 hour for income in the last week, or in an unpaid role as a family member for more than 18 hours?” Of the total 7,992 people who participated in the survey, 4,352 were women and 1,815 of whom were women aged 19 years or older (Figure 1).

In this study, women were considered daytime workers if they answered “yes” to the following question: “Do you usually work between 6 AM and 6 PM?” Women were considered shift workers if they worked any of the following: evening shifts (2 AM to midnight), night shifts (9 PM to 8 AM the next day), regular or irregular day and night shifts, 24-hour shifts, and other alternatives to a standard daytime work schedule. Of the 1,815 working women aged 19 years or older, 1,493 were daytime workers and 322 were shift workers.

### Variables

#### Depression

Depression in the 7th KNHANES was measured using the Patient Health Questionnaire-9 (PHQ-9) [16]. The PHQ-9 consists of nine questions for determining if respondents have suffered from depression-related symptoms over the previous 2 weeks, for which there are four possible responses: ‘not at all,’ ‘several days,’ ‘more than half the days,’ or ‘nearly every day.’ The PHQ-9 results are calculated by summing scores for each question (0 not at all, to 3 nearly every day). Higher scores indicate a greater degree of depression. A score of 0-4 points indicates minimal depression, 5-9 points indicates mild depression, 10-14 points indicates moderate depression, 15-19 points indicates moderately severe depression, and 20-27 points indicates severe depression. For analysis, 10 points was treated as the cutoff, with summed scores of 10 or greater indicating depression.

#### Health-related behaviors

Health-related behaviors were drinking status, smoking status, frequency of aerobic physical activity, and average nightly sleeping hours for both weeknights and weekend nights.

Participants were considered non-drinkers if they had not consumed alcohol in the past month, and drinkers if they had. They were considered non-smokers if they did not smoke or had quit smoking, and smokers if they smoked daily or occasionally. Respondents were considered physically inactive if they participated in aerobic physical activity rarely or never; physically active meant they participated in aerobic physical activity daily or often. Average hours slept for both weeknights and weekend nights were classified in four categories: 4 hours or less, 5–6 hours, 7–8 hours, or 9 hours or more [17,18].

#### Use of medical services

To determine respondents’ use of medical services, KNHANES collected data on the following: Participants answered ‘yes’ or ‘no’ to questions asking whether they had received a medical check-up and cancer screening within the previous 2 years and a flu vaccination within the previous year. Unmet medical needs and unmet dental needs were also determined.

#### General characteristics

General characteristics included age, education level, personal income, marital status, household type, relationship with other household members, occupation, average weekly working hours, and subjective health status.

Age was classified according to the following five groups: 19–29, 30–39, 40–49, 50–59, or 60 years and older. Education level was classified as a middle school level or less, high school level, or college level and above. Income was divided into five classifications according to the quintile classification criteria of the KNHANES : high, upper middle, middle, lower middle, and low. Marital status was either married or unmarried, and household types were divided into single-person and multi-person. Job type was classified as managers/professionals, office/service/sales, or other. The number of working hours per week was classified as less than 40 hours, 40 hours, between 40 and 52 hours, and more than 52 hours, based on labor law. Subjectively perceived health status was classified as good, moderate, or poor.

### Data collection

The data used for this study were downloaded from the KNHANES website. The KNHANES consists of a self-reported health survey, check-up survey, and nutrition survey at household level, taking place over a 3-year period. Tests were conducted at mobile screening centers, and nutritional surveys were conducted by visiting target households in person. The Korea Centers for Disease Control and Prevention publishes the results of the survey with the disclosure of raw data on the KNHANES website, which only provides anonymous data so that individuals cannot be identified from the survey data in compliance with the Personal Information Protection Act and Statistics Act.

### Data analysis

Data analysis in this study was conducted using IBM SPSS ver. 20.0 (IBM Corp., Armonk, NY, USA) and complex-sample analysis, considering the complex-sample elements, strata, cluster, and weight. The specific data analysis methods were as follows.

(1) The general characteristics, health-related behaviors, and use of medical services among women daytime and shift workers were analyzed using complex-sample statistics. Depression was quantified using the mean and standard error with complex-sample descriptive analysis.

(2) A comparison of the general characteristics, health-related behaviors, and use of medical services between women daytime and shift workers was conducted using the complex-sample t-test and cross-tab analysis.

(3) Differences in the prevalence and intensity of depression between women daytime workers and shift workers were analyzed using a complex-sample general linear model.

(4) The general characteristics, health-related behaviors, and differences in the prevalence and intensity of depression according to the use of medical services were analyzed using a complex-sample general linear model.

(5) The depression-related factors of women daytime workers and shift workers were analyzed using a complex-sample general linear model.

## Results

### Differences in general characteristics between women daytime and shift workers

Women daytime and shift workers differed in terms of age, education level, personal income, marital status, and average hours worked per week. Among the age ranges, the smallest proportion of daytime workers was 19 to 29 years old (16.2%), as opposed to shift workers, for whom women aged 19 to 29 years made up the largest proportion (30.6%), showing a statistically significant difference (χ^2^=43.97, *p*<.001). There was also a difference in education level, with 46.8% of daytime workers reporting college education or higher, whereas 50.3% of shift workers reporting having graduated from high school (χ^2^=45.56, *p*<.001). For personal income, the highest proportion of daytime workers reported having upper middle (21.6%) or high (22.1%) personal income levels, while the highest proportion of shift workers reported having low (21.3%) and lower middle (25.8%) personal income levels (χ^2^=16.85, *p*=.030). There were also more unmarried shift workers (35.6%) than their daytime working counterparts (20.1%) (χ^2^=45.56, *p*<.001). In terms of average hours worked per week, a higher proportion of daytime workers reported working 40 hours per week or between 40 and 52 hours per week than shift workers, who mostly worked fewer than 40 hours per week (χ^2^=69.94, *p*<.001) (Table 1).

### Differences in health-related behaviors, use of health care services, and depression between women daytime and shift workers

Daytime and shift working women differed in terms of drinking status, smoking status, physical activity, average hours slept per night on weeknights, medical check-ups, and cancer screening. Shift workers had a higher proportion of drinkers (58.3%) than daytime workers (50.6%) (χ^2^=6.49, *p*=.032). In addition, shift workers had a higher proportion of smokers (15.0%) than daytime workers (6.1%) (χ^2^=30.79, *p*<.001). Shift workers also engaged in physical activity (48.5%) less often than daytime workers (40.1%) (χ^2^=7.87, *p*=.025). Daytime workers averaged 7-8 hours of sleep on weeknights (54.3%) more often than shift workers (49.8%), and more shift workers reported sleeping fewer than 5 hours per weeknight (8.3%) than daytime workers (3.9%) (χ^2^=14.17, *p*=.024). A lower proportion of shift workers received a medical check-up within the previous 2 years (60.9%) than daytime workers (73.6%) (χ^2^=21.85, *p*<.001). Fewer shift workers received cancer screening (54.3%) than daytime workers (64.5%) (χ^2^=12.38, *p*<.001).

The average scores for depression were higher among shift workers (3.19 points) than among daytime workers (2.42 points) (t=2.85, *p*=.005). A higher proportion of shift workers also scored more than 10 points (indicating moderate depression) on the PHQ-9 (8.6%) than daytime workers (χ^2^=14.22, *p*=.001) (Table 2).

### Differences in depression according to general characteristics, health-related behaviors, and use of healthcare services among daytime workers

Daytime workers showed different results for depression according to age, personal income, marital status, household type, health status, drinking status, smoking status, physical activity, average hours slept per night on weeknights, average hours slept per night on weekend nights, medical check-up status, cancer screening status, unmet health care needs, and unmet dental care needs.

Depression was higher in women aged 19 to 29 years than in women aged 60 years and over (F=6.88, *p*<.001), and women with a high level of personal income showed the lowest level of depression (F=6.95, *p*<.001). Single women more frequently had depression than married women (F=18.66, *p*<.001), and women from single-person households had higher levels of depression than women from multi-person households (F=6.24, *p*=.013). Compared to women who perceived their health as being good, women who perceived having moderate or poor normal or bad health had higher levels of depression (F=48.24, *p*<.001). Women who drank alcohol also had higher scores for depression than women who did not drink (F=10.11, *p*=.002), and women who smoked had higher depression levels than non-smoking women (F=14.24, *p*<.001). Women who were physically active were more depressed than women who were inactive (F=6.10, *p*=.015). In addition, women who slept fewer than 5 hours on average during the week had higher levels of depression (F=6.80, *p*<.001), as were women who slept fewer than 5 hours on average during the weekend (F=4.07, *p*=.008). Women who had not received a medical check-up (F=7.74, *p*=.009) or cancer screening (F=6.18, *p*=.014) within the previous 2 years were more depressed on average. Women daytime workers with unmet medical needs (F=28.18, *p*<.001) and unmet dental care needs (F=11.46, *p*=.001) also showed higher levels of depression (Table 3).

### Differences in depression according to general characteristics, health-related behaviors, and use of healthcare services among shift workers

Depression among women shift workers varied depending on age, personal income, marital status, health status, smoking status, cancer screening status, unmet health care needs, and unmet dental care needs.

Depression was higher in women aged 19 to 29 and 30 to 39 compared to the other age ranges (F=4.12, *p*=.003). Women with a high level of personal income had the lowest level of depression (F=6.17, *p*<.001). Single women had higher levels of depression than married women (F=6.68, *p*=.011). Women who smoked showed higher levels of depression than women who did not smoke (F=11.31, *p*=.001). Compared to women who perceived their health as being good, women who perceived their health as normal or bad had higher levels of depression (F=14.62, *p*<.001). Shift work women who had not received a cancer screening (F=4.06, *p*=.046) were more depressed, and depression was high among women with unmet health care needs (F=5.59, *p*=.019) and unmet dental care needs (F=6.01, *p*=.015) (Table 3).

### Factors related to depression in women workers

Depression-related factors for women daytime workers were age, personal income, health status, drinking status, smoking status, average hours slept per night, and unmet medical care needs, with 26% model explanation. Depression was higher in women aged 19 to 29 years than in women aged 60 years or older. Lower personal income and poor perceived health status also corresponded to higher levels of depression. Women who drank alcohol and smoked had higher levels of depression than non-drinking and non-smoking women. Depression was higher among women who slept on average fewer than 5 hours per night on weeknights compared to women who averaged between 7 and 9 hours of sleep on weeknights. Depression was also higher among women with unmet medical care needs (Table 4).

As for women shift workers, depression-related factors included age, personal income, health status, smoking status, unmet health care needs, and unmet dental care needs; the model including these factors explained 31% of variance. Compared to women aged 60 years or older, depression was higher among women aged 30 to 39 years. Lower personal income and poor perceived health status corresponded to higher levels of depression. Women who smoked experienced higher levels of depression than non-smoking women, and depression was also higher among women with unmet medical and dental care needs (Table 5).

## Discussion

In this study, depression was higher among women shift workers than among their daytime working counterparts. This is consistent with the results of a study of employed women showing a higher rate of depression among women who performed shift work than among women who were daytime workers [19] and a study of male and female workers that also showed a higher rate of depression among shift workers than among daytime workers [8,20]. Considering that the rate of depression is higher among women workers than men [20,21], these results indicate that women who perform shift work experience the most depression among various categories of workers. Because shift work is different from the 24-hour biological rhythm [22], it interferes with workers’ biological rhythm, leading to a variety of physical health problems as well as various mental health problems such as anxiety and depression [23,24]. However, shift work is inevitably performed due to industrial development and flexible working hours [8]. Therefore, efficient and practical interventions for depression prevention and management are needed for women shift workers, and to do so, it is necessary to accumulate evidence through repeated studies that seek to identify the factors related to their experience of depression. In addition, the prevalence of shift work has continued to increase and is expected to further increase in the future [25,26]. This requires community-level and national attention and flexible measures such as adjusting shift work cycles, reducing shift work hours, and ensuring sufficient rest during shift work [8].

This study identified common influential factors on depression for women workers: age, personal income, perceived health status, smoking status, and unmet medical needs. For daytime working women drinking status and average hours slept per weeknight were additional factors; whereas unmet dental needs were an extra factor for shift working women.

Age affected depression differently, however, between daytime and shift workers, with daytime workers experiencing higher levels of depression in the 19–29 years age range than in the 60 years and older range, while shift workers experienced higher levels of depression in the 30–39 years range than in the 60 years and older range. This is somewhat consistent with the results of a study on paid workers that showed higher rates of depression among younger workers, especially those between 19 and 39 years old [27]. The high level of depression among workers aged 19 to 39 years compared to other age ranges reflects the socioeconomic environment of South Korea, where there has been an increase in youth unemployment, nontraditional employment, and job insecurity [27]. In addition, people in their 30s often experience a new family environment due to getting married and having children, which can lead to conflicts between work and home roles, and possibly deepen depression [21]. This suggests that depression intervention and prevention programs for women workers need to focus more on those in their 20s and 30s. In addition, differences were observed between daytime and shift working women in terms of which age groups experienced the most depression, so further research is needed.

The analysis of personal income related to depression showed that lower personal income for both daytime and shift workers corresponded to higher levels of depression. This is consistent with previous studies which have found that lower income groups experience higher levels of depression [27-29]. In addition, this study found differences in depression levels according to personal income were greater among shift workers than among daytime workers. These findings support the need for depression prevention and management programs for women workers to target low-income workers, with a particular focus on shift workers with low income.

The analysis of smoking status found that women who smoked showed higher rates of depression than non-smokers among both daytime workers and shift workers, while women who drank alcohol showed a higher rate of depression than non-drinking women only among daytime workers. Prior studies have shown that women who smoked and drank alcohol had higher levels of depression than non-smoking women and non-drinking women [28,30]. However, some studies have found no link between smoking or drinking and depression, reflecting the difficulty of determining the factors that influence the decision to smoke or drink as they relate to depression [28]. In this study, drinking had an influence on depression only among daytime work women, unlike previous studies. Therefore, greater evidence should be accumulated through additional research on the influence of smoking and drinking on depression among women workers. In addition, age at first exposure to smoking or drinking, the degree of exposure, and the effects of smoking or drinking, as well as current smoking and drinking status, can affect depression [30]. Therefore, more in-depth understanding of issues related to smoking and drinking is required, such as how long women have been regular smokers/drinkers, their regular amount of smoking/drinking, and the frequency with which they smoke/drink.

Among women with unmet medical needs, the rate of depression was high for both daytime workers and shift workers, while for women with unmet dental needs, the rate of depression was high only for shift workers. Unmet health care needs lead to a higher likelihood of negative health-related consequences [31], and if one has unmet health care needs, there is also a strong possibility of having unmet dental care needs [32]. The relationship between mental health and unmet health care needs is strong [31], and the high rate of depression among female daytime and shift workers with unmet medical needs in this study can be understood in this context. In addition, if the demand for health services is high, even if people are provided consistent health services, they might not perceive those services as having been sufficient [32]. It has been found that one reason for the high rate of unmet dental care needs is that those with high income feel that dental care is a low priority compared to other problems, while those with low income cannot always afford dental care [33]. Given the high proportion of female shift workers with a low income in this study, unmet dental needs were likely related to depression only for shift workers because of their inability to afford sufficient dental care or lack of desire to obtain dental care due to economic strain. However, this study did not identify the reasons for unmet dental needs, so it is necessary to identify both the presence of unmet dental needs and the reasons for unmet dental needs in future studies.

The number of average hours slept on weeknights was related to depression only among female daytime workers, and depression was high among women who slept fewer than 5 hours on average per night. This can be understood in the same context as prior studies finding that workers who slept for fewer than 7 hours per night on average had a 2.16 times higher incidence of depression [20], and that fewer hours of sleep resulted in a higher degree of depression [27]. Sleep and depression are closely related [25], and sleep disorders can lead to depression [19]. Therefore, it is important to provide interventions for the prevention and management of depression among women workers that consider time spent sleeping and factors related to sleep disorders. In addition, the relationship between time spent sleeping and depression was significant only among daytime workers, which could be understood in relation to the results of a study [25] finding that shift nurses had adapted to irregular sleep patterns, while daytime nurses were accustomed to regular sleep. However, for shift workers, the risk of sleep disorders is higher than that of daytime workers, so it is necessary to conduct further studies on the relationship between sleep and depression according to type of work. In addition, it is necessary to identify sleep-related characteristics such as sleep quality and time spent sleeping, to determine their relationship with depression.

In this study, women daytime and shift workers differed in age, education, personal income, marital status, and time spent working. The lowest proportion of daytime workers were ages 19–29 years, while the highest proportion of shift workers were ages 19–29 years, indicating that the typical age of women shift workers was lower than that of daytime workers. This can be understood in the context of the results of prior studies [8,26] that showed that shift workers tended to be younger than daytime workers. A prior study on nurses found that most new (younger) nurses are assigned to perform shift work, while experienced nurses are assigned to daytime work [26]. In general, workers prefer daytime work over shift work and is likely that the phenomenon of assigning shift work to new employees is also true of other professions besides nursing, potentially explaining the lower average age of women shift workers than daytime workers.

There was a difference in education level, as the largest share of women daytime workers were college graduates, while the largest share of shift workers were high school graduates. This is consistent with prior studies [8,20] that reported that daytime workers tended to have a higher education level than shift workers. Considering previous studies that found that a high percentage of non-regular workers performed shift work [34] and that shift workers had a low average education level [8], this study’s results can also be understood in this context. While this difference may be due to the characteristics of daytime and shift work, our analysis did not take that possibility into account and may be an area for future research to clarify.

Personal income was lower and lower education levels were found for women shift workers than for daytime workers. A prior study reported that daytime workers tended to have a higher economic status than shift workers, but there was no statistical significance [20], which differed from this study. Education levels are related to income levels, and low education levels tend to correspond to low income levels [18,28]. In addition, this study found that the average weekly time spent working tended to be lower for shift workers than for daytime workers, which is also related to low income levels.

There was a higher proportion of unmarried women among shift workers than among daytime workers. This is consistent with previous studies [20,23] that found that there were more single women among shift workers than among daytime workers. This is likely because married women often have to balance work and family, which can lead to many problems if they perform shift work. Thus, married women likely tend to avoid shift work more than unmarried women. In addition, shift work tends to be assigned to new employees, and experienced employees are assigned daytime work [19]. Therefore, considering that women tend to get married after being employed rather than getting married and then seeking employment, new employees are more likely to be unmarried and are thus also more likely to be assigned shift work.

Drinking and smoking were more prevalent among shift workers than daytime workers. This is inconsistent with a preceding study that found no difference in the prevalence of drinking and smoking between daytime and shift workers [8]. This finding can likely be attributed to the younger age of women shift workers compared to daytime workers in this study. Prior studies on workers’ drinking and smoking habits have analyzed differences according to occupation type, such as manufacturing jobs, office jobs, and service jobs, and found differences in the drinking and smoking habits of workers across different occupations [35,36]. However, differences in drinking and smoking habits between daytime workers and shift workers are measured differently depending on the study, so further studies are needed. In addition, Korean culture emphasizes drinking as an important activity for social relationships and as an opportunity to exchange information with co-workers and bosses, so it is necessary to analyze both the prevalence of drinking and the reasons for drinking. It has also been found that the risk of smoking is high among night shift workers [29], meaning that it is also necessary to consider the type of shift when analyzing the smoking rate among shift workers.

On average, women daytime workers often slept for the recommended number of hours per weeknight, while shift workers often slept on average for fewer than 5 hours per weeknight. Prior studies, however, have found that there was no difference in average time spent sleeping between daytime workers and shift workers [8] or that the total time spent sleeping by shift workers was higher than daytime workers [25]. Thus, it is necessary to conduct further studies on the average time spent sleeping by women daytime workers and shift workers. In addition, shift work disrupts biorhythm, causing irregular sleep patterns and reducing sleep quality [24,25], and a prior study reported that shift workers experienced irregular sleep times, took more sleeping pills, and experienced a lower quality of sleep than daytime workers [25]. Therefore, identifying the qualitative characteristics of sleep as well as quantitative characteristics (e.g., time spent sleeping) may help improve sleep issues according to daytime and shift work in women.

In this study, the use of health services between women daytime workers and shift workers differed for medical check-ups and cancer screening, and the percentage of shift workers who received a medical check-up and cancer screening within the previous 2 years was lower than that of daytime workers. This is partly consistent with a previous study [29] that reported that the rate of medical check-ups was lower among women who worked at night compared to women who worked during the day, but that there was no difference in cancer screening rates. Considering that the proportion of non-regular workers is high for shift work and the health insurance subscription rate is low for non-regular workers [29], this result can likely be attributed to the high possibility that women who perform shift work are less likely to receive health insurance benefits than women who perform daytime work. In addition, this study’s finding that women shift workers were younger, have less education, and have a lower income than daytime workers likely also relates to differences in health insurance benefits and thus the rate at which shift workers attend medical check-ups and cancer screenings. Since shift work interference with biological rhythm can lead to a variety of health problems [22,24], the need for early detection and treatment of health problems through medical check-ups and cancer screenings is greater for shift working women than for daytime workers. Closely examining the reasons why women shift workers are less likely to have medical check-ups and cancer screenings may be helpful for policies to narrow this gap.

This study has the following limitations. All types of work other than daytime work were classified as shift work, and thus, the various contexts of shift work could not be considered. In addition, job-related characteristics, which can affect depression among working women, were not available. Future studies should examine different types of shift work to identify differences according to type of work, with appropriate consideration of a wider range of variables that may also affect depression among working women.

In summary, depression was found to be higher among women who performed shift work than among women who performed daytime work. The factors related to depression among women daytime workers were age, personal income, perceived health status, smoking status, drinking status, average hours of sleep per weeknight, and unmet medical needs, while those related to depression among women shift workers were age, personal income, perceived health status, smoking status, unmet medical needs, and unmet dental needs. Therefore, it is necessary to provide practical and efficient evidence-based interventions for depression prevention and management among women who perform shift work, taking into account depression-related factors. In addition, national support and various policies that specifically support women performing shift work is needed to improve their health and well-being.

## Figures and Tables

**Table 1.** General characteristics of daytime and shift working women (N=1,815)

Characteristics	Categories	Daytime workers (n=1,493)		Shift workers (n=322)		χ^2^ (*p*)
		n^†^	%^‡^(SE)	n^†^	%^‡^(SE)	
Age (year)	19–29	173	16.2 (1.3)	75	30.6 (3.2)	43.97 (<.001)
	30–39	246	18.9 (1.5)	40	12.8 (1.9)	
	40–49	349	23.0 (1.4)	77	23.2 (3.0)	
	50–59	368	24.3 (1.5)	80	22.3 (2.8)	
	≥60	357	17.6 (1.4)	50	11.1 (1.8)	
Education level	≤Middle school	405	22.3 (1.7)	61	16.1 (2.2)	45.56 (<.001)
	High school	458	31.0 (1.5)	148	50.3 (3.2)	
	≥College	630	46.8 (1.8)	113	33.6 (3.1)	
Personal income	Low	238	17.9 (1.2)	59	21.3 (3.2)	16.85 (.030)
	Lower middle	284	17.9 (1.3)	79	25.8 (3.2)	
	Middle	300	20.5 (1.3)	57	16.7 (2.4)	
	Upper middle	328	21.6 (1.4)	66	19.4 (2.8)	
	High	340	22.1 (1.4)	60	16.8 (2.4)	
Marital status	Married	1,259	79.9 (1.5)	230	64.4 (3.2)	37.47 (<.001)
	Unmarried	234	20.1 (1.5)	92	35.6 (3.2)	
Household type	Single-person	165	10.6 (1.2)	49	13.5 (2.1)	2.47 (.165)
	Multi-person	1,328	89.4 (1.2)	273	86.5 (2.1)	
Household head	Self	554	35.8 (1.8)	129	36.8 (2.9)	0.12 (.746)
	Other	939	64.2 (1.8)	193	63.2 (2.9)	
Occupation type	Manager/professional	342	25.6 (1.4)	96	26.5 (2.8)	4.77 (.236)
	Office/service/sales	713	49.5 (1.5)	159	54.0 (3.5)	
	Others	438	25.0 (1.6)	67	19.4 (2.4)	
Paid worker	Yes	1147	79.0 (1.3)	229	75.2 (2.9)	2.35 (.210)
	No	346	21.0 (1.3)	93	24.8 (2.9)	
Time spent working (hour/week)	≤39	650	41.8 (1.7)	213	66.3 (2.9)	69.94 (<.001)
	40	321	22.0 (1.3)	25	9.4 (1.9)	
	41–51	331	23.9 (1.4)	53	15.2 (2.1)	
	≥52	191	12.4 (1.0)	31	9.2 (1.7)	
Perceived health status	Poor	245	16.0 (1.2)	52	16.4 (2.3)	0.65 (.800)
	Moderate	798	52.7 (1.5)	179	54.5 (3.2)	
	Good	450	31.3 (1.3)	91	29.1 (3.0)	

† Unweighted and valid frequency,

‡ valid percentage.

**Table 2.** Health behaviors and use of health services of women daytime and shift workers (N=1,815)

Characteristics	Categories	Daytime workers (n=1,493)		Shift workers (n=322)		χ^2^ (*p*) or t (*p*)		
		n^†^ or range	%^‡^ (SE) or mean±SE	n^†^ or range	%^‡^ (SE) or mean±SE			
Health behavior								
Drinking	Yes	703	50.6 (1.6)	175	58.3 (3.1)	6.49 (.032)		
	No	788	49.4 (1.6)	146	41.7 (3.1)			
Smoking	Yes	74	6.1 (0.9)	43	15.0 (2.4)	30.79 (<.001)		
	No	1,416	93.9 (0.9)	279	85.0 (2.4)			
Physical activity	Yes	583	40.1 (1.5)	147	48.5 (3.3)	7.87 (.025)		
	No	909	59.9 (1.5)	174	51.5 (3.3)			
Time spent sleeping (hour/weeknight)	≤4	52	3.9 (0.6)	28	8.3 (1.8)	14.17 (.024)		
	5–6	512	35.2 (1.5)	110	33.4 (2.8)			
	7–8	817	54.3 (1.5)	159	49.8 (3.3)			
	≥9	109	6.7 (0.8)	25	8.4 (2.0)			
Time spent sleeping (hour/weekend night)	≤4	24	1.6 (0.4)	12	3.2 (1.0)	3.63 (.477)		
	5–6	273	17.4 (1.1)	67	17.0 (2.2)			
	7–8	806	52.7 (1.4)	165	51.4 (3.3)			
	≥9	387	28.3 (1.5)	78	28.5 (3.4)			
Health service use								
Medical check-up	No	377	26.4 (1.3)	121	39.1 (3.4)	21.85 (<.001)		
	Yes	1,115	73.6 (1.3)	201	60.9 (3.4)			
Cancer screening	No	490	35.5 (1.6)	136	45.7 (3.6)	12.38 (<.001)		
	Yes	1,002	64.5 (1.6)	186	54.3 (3.6)			
Influenza vaccination	No	890	61.6 (1.6)	202	66.9 (3.0)	3.32 (.113)		
	Yes	601	38.4 (1.6)	120	33.1 (3.0)			
Unmet medical needs	No	1,331	89.2 (1.0)	293	90.8 (1.7)	0.71 (.448)		
	Yes	162	10.8 (1.0)	29	9.2 (1.7)			
Unmet dental needs	No	1,048	71.5 (1.4)	228	71.0 (3.0)	0.04 (.879)		
	Yes	443	28.5 (1.4)	94	29.0 (3.0)			
Depression	Total	0–27	2.42±0.10	0–23	3.19±0.27	2.85 (.005)		
	Yes ( ≥10)	56	3.8 (0.6)	29	8.6 (1.8)	14.22 (.001)		
	No (<10)	1,430	96.2 (0.6)	293	91.4 (1.8)			

† Unweighted and valid frequency,

‡ valid percentage.

**Table 3.** Depression according to general characteristics, health behaviors, and use of health services (N=1,815)

Characteristics	Categories	Daytime workers (n=1,493)		Shift workers (n=322)	
		Mean (SE)	F (*p*)	Mean (SE)	F (*p*)
Age (year)	19–29	3.72 (0.29)	6.88 (<.001)	3.68 (0.56)	4.12 (.003)
	30–39	2.20 (0.22)		4.95 (0.78)	
	40–49	2.14 (0.16)		2.93 (0.52)	
	50–59	2.15 (0.21)		2.42 (0.39)	
	≥60	2.21 (0.20)		1.89 (0.47)	
Education level	≤Middle school	2.55 (0.21)	0.43 (.649)	2.96 (0.65)	0.22 (.805)
	High school	2.31 (0.16)		3.35 (0.39)	
	≥College	2.43 (0.14)		3.05 (0.38)	
Personal income	Low	2.83 (0.24)	6.95 (<.001)	3.54 (0.68)	6.17 (<.001)
	Lower middle	2.94 (0.27)		4.46 (0.64)	
	Middle	2.58 (0.19)		2.90 (0.53)	
	Upper middle	2.16 (0.16)		2.86 (0.47)	
	High	1.76 (0.15)		1.58 (0.29)	
Marital status	Married	2.17 (0.10)	18.66 (<.001)	2.61 (0.30)	6.68 (.011)
	Unmarried	3.42 (0.27)		4.24 (0.53)	
Household type	Single-person	3.25 (0.35)	6.24(.013)	3.79 (0.52)	1.44 (.233)
	Multi-person	2.32 (0.10)		3.10 (0.29)	
Household head	Self	2.66 (0.16)	3.35 (.069)	3.54 (0.39)	1.17 (.282)
	Other	2.83 (0.12)		2.99 (0.35)	
Occupation type	Manager/professional	2.39 (0.19)	0.34 (.714)	2.95 (0.46)	0.25 (.781)
	Office/service/sales	2.49 (0.14)		3.37 (0.40)	
	Others	2.30 (0.19)		3.04 (0.59)	
Paid worker	Yes	2.38 (0.10)	0.72 (.396)	3.11 (0.30)	0.31 (.582)
	No	2.56 (0.19)		3.43 (0.51)	
Time spent working	≤39	2.31 (0.14)	0.92 (.432)	3.04 (0.31)	0.55 (.652)
(hour/week)	40	2.31 (0.19)		2.82 (1.06)	
	41–51	2.73 (0.23)		3.35 (0.49)	
	≥52	2.37 (0.25)		4.41 (1.14)	
Perceived health status	Bad	4.81 (0.34)	48.24 (<.001)	6.01 (0.76)	14.62 (<.001)
	Normal	2.28 (0.13)		3.07 (0.37)	
	Good	1.45 (0.12)		1.82 (0.30)	
Drinking status	Yes	2.70 (0.14)	10.11 (.002)	3.59 (0.40)	3.12 (.079)
	No	2.14 (0.12)		2.63 (3.34)	
Smoking status	Yes	4.83 (0.67)	14.24 (<.001)	6.06 (0.96)	11.31 (.001)
	No	2.27 (0.09)		2.68 (0.24)	
Physical activity	Yes	2.69 (0.15)	6.10 (.015)	3.38 (0.39)	0.55 (.461)
	No	2.24 (0.12)		3.01 (0.34)	
Time spent sleeping (hour/weeknight)	<5	6.03 (0.82)	6.80 (<.001)	4.34 (1.24)	2.27 (.082)
	5–6	2.20 (0.16)		3.41 (0.45)	
	7–8	2.28 (0.11)		2.63 (0.28)	
	≥9	2.51 (0.30)		4.54 (0.81)	
Time spent sleeping (hour/weekend night)	<5	6.28 (1.29)	4.07 (.008)	4.57 (1.56)	0.50 (.686)
	5–6	2.25 (0.22)		3.28 (0.68)	
	7–8	2.22 (0.12)		2.94 (0.39)	
	≥9	2.66 (0.19)		3.43 (0.47)	
Medical check-up	No	2.84 (0.19)	7.74 (.009)	3.86 (0.49)	3.76 (.054)
	Yes	2.27 (0.11)		2.76 (0.29)	
Cancer screening	No	2.72 (0.17)	6.18 (.014)	3.76 (0.43)	4.06 (.046)
	Yes	2.23 (0.11)		2.71 (0.30)	
Influenza vaccination	No	2.49 (0.12)	0.87 (.351)	3.42 (0.35)	2.08 (.152)
	Yes	2.31 (0.15)		2.72 (0.35)	
Unmet medical needs	No	2.18 (0.09)	28.18 (<.001)	2.94 (0.25)	5.59 (.019)
	Yes	4.37 (0.41)		5.69 (1.14)	
Unmet dental needs	No	2.20 (0.10)	11.46 (.001)	2.76 (0.28)	6.01 (.015)
	Yes	2.97 (0.21)		4.25 (0.56)	

**Table 4.** Factors related to depression among women daytime workers (N=1,493)

Characteristics	Categories	B (SE)	95% CI		t	*p*
			Lower	Upper		
Constant		0.02 (0.26)	–0.50	0.54	0.08	.937
Age (year)^†^	19–29	1.42 (0.40)	0.63	2.21	3.57	<.001
	30–39	0.52 (0.30)	–0.06	1.1	1.76	.081
	40–49	0.33 (0.23)	–0.12	0.78	1.47	.145
	50–59	0.22 (0.22)	–0.22	0.66	0.97	.334
Personal income^†^	Low	0.57 (0.26)	0.06	1.08	2.2	.029
	Lower middle	0.60 (0.27)	0.08	1.13	2.27	.025
	Middle	0.48 (0.22)	0.05	0.91	2.19	.030
	Upper middle	0.12 (0.22)	–0.24	0.61	0.88	.380
Marital status^†^	Unmarried	0.54 (0.33)	–0.12	1.19	1.62	.108
Household type^†^	One-person	0.46 (0.32)	–0.18	1.09	1.41	.160
Perceived health status^†^	Bad	2.99 (0.33)	2.35	3.63	9.19	<.001
	Normal	0.67 (0.15)	0.38	0.96	4.56	<.001
Drinking status^†^	Yes	0.33 (0.16)	0.02	0.63	2.1	.037
Smoking status^†^	Yes	1.59 (0.59)	0.42	2.75	2.69	.008
Physical activity^†^	Yes	0.25 (0.14)	–0.04	0.53	1.72	.087
Time spent sleeping^†^ (hour/weeknight)	<5	2.47 (0.88)	0.74	4.2	2.82	.005
	5–6	–0.06 (0.17)	–0.40	0.28	–0.34	.735
	≥9	–0.13 (0.32)	–0.77	0.5	–0.41	.679
Time spent sleeping^†^ (hour/weekend night)	<5	0.73 (1.20)	–1.64	3.1	0.61	.543
	5–6	0.07 (0.24)	–0.40	0.54	0.31	.760
	≥9	0.08 (0.20)	–0.32	0.48	0.4	.691
Medical check-up^†^	No	0.18 (0.27)	–0.35	0.71	0.66	.508
Cancer screening^†^	No	–0.34 (0.22)	–0.77	0.09	–1.55	.122
Unmet medical needs^†^	Yes	1.33 (0.36)	0.63	2.03	3.75	<.001
Unmet dental needs^†^	Yes	0.17 (0.18)	–0.20	0.53	0.91	.367
		R^2^=.26, F=11.40, *p*<.001				

CI: Confidence interval.

† The reference groups for dummy variables were as follows: age (≥60 years), personal income (high), marital status (married), household type (multi-person), perceived health status (good), drinking status (no), smoking status (no), physical activity (no), time spent sleeping (7–8 hours), medical check-up (yes), cancer screening (yes), unmet medical needs (no), and unmet dental needs (no).

**Table 5.** Factors related to depression among women shift workers (N=322)

Characteristics	Categories	B (SE)	95% CI		t	*p*
			Lower	Upper		
Constant		–1.26 (0.71)	–2.66	0.13	–1.79	.076
Age (year)^†^	19–29	1.26 (1.13)	–0.98	3.49	1.11	.268
	30–39	1.94 (0.83)	0.29	3.58	2.33	.021
	40–49	0.77 (0.59)	–0.40	1.94	1.3	.196
	50–59	0.06 (0.64)	–1.21	1.32	0.09	.927
Personal income^†^	Low	1.15 (0.58)	0.01	2.29	1.99	.049
	Lower middle	2.24 (0.61)	1.05	3.44	3.7	<.001
	Middle	1.29 (0.56)	0.18	2.4	2.3	.023
	Upper middle	1.10 (0.52)	0.07	2.14	2.1	.037
Marital status^†^	Unmarried	0.90 (0.85)	–0.78	2.58	1.06	.29
Perceived health status^†^	Bad	3.08 (0.67)	1.77	4.4	4.63	<.001
	Normal	1.19 (0.43)	0.35	2.04	2.81	.006
Smoking status^†^	Yes	2.07 (0.86)	0.37	3.76	2.4	.017
Cancer screening^†^	No	0.07 (0.45)	–0.82	0.97	0.16	.870
Unmet medical needs^†^	Yes	2.90 (0.92)	1.09	4.7	3.17	.002
Unmet dental needs^†^	Yes	1.11 (0.52)	0.08	2.14	2.13	.035
		R^2^=.31, F=4.66, *p*<.001				

CI: Confidence interval.

† The reference groups for dummy variables were as follows: age (≥60 years), personal income (high), marital status (married), perceived health status (good), smoking status (no), cancer screening (yes), unmet medical needs (no), and unmet dental needs (no).
